# Artesunate inhibits the mevalonate pathway and promotes glioma cell senescence

**DOI:** 10.1111/jcmm.14717

**Published:** 2019-11-20

**Authors:** Shupei Wei, Lili Liu, Zhiyu Chen, Wenli Yin, Yingzi Liu, Qianying Ouyang, Feiyue Zeng, Yingjie Nie, Tao Chen

**Affiliations:** ^1^ The First Affiliated Hospital of Guangzhou Medical University Guangzhou China; ^2^ People's Hospital of Rizhao Rizhao China; ^3^ Clinical Research Lab Center Guizhou Provincial People's Hospital Guiyang China; ^4^ Xiangya Hospital Central South University Changsha China

**Keywords:** artesunate, distant seeding, glioma, mevalonate pathway, senescence

## Abstract

Glioma is a common brain malignancy for which new drug development is urgently needed because of radiotherapy and drug resistance. Recent studies have demonstrated that artemisinin (ARS) compounds can display antiglioma activity, but the mechanisms are poorly understood. Using cell lines and mouse models, we investigated the effects of the most soluble ARS analogue artesunate (ART) on glioma cell growth, migration, distant seeding and senescence and elucidated the underlying mechanisms. Artemisinin effectively inhibited glioma cell growth, migration and distant seeding. Further investigation of the mechanisms showed that ART can influence glioma cell metabolism by affecting the nuclear localization of SREBP2 (sterol regulatory element‐binding protein 2) and the expression of its target gene HMGCR (3‐hydroxy‐3‐methylglutaryl coenzyme A reductase), the rate‐limiting enzyme of the mevalonate (MVA) pathway. Moreover, ART affected the interaction between SREBP2 and P53 and restored the expression of P21 in cells expressing wild‐type P53, thus playing a key role in cell senescence induction. In conclusion, our study demonstrated the new therapeutic potential of ART in glioma cells and showed the novel anticancer mechanisms of ARS compounds of regulating MVA metabolism and cell senescence.

## 1 INTRODUCTION

Glioma, for which radiotherapy effects are limited due to aberrant radioresistance,^1^ is the most common and primary brain malignancy worldwide.^2^ Therefore, the exploration of novel chemotherapeutics or agents is urgently needed for the future treatment of glioma.

Artemisinin (ARS) analogues, which are all derived from the *Artemesia annua L* herb (also known as sweet wormwood), are well known for their effective application in antimalarial pharmacotherapy.^3^ Recent studies have shown that ARS compounds show promising tumouricidal activity, as they exert antiangiogenic and proapoptotic effects and inhibit growth, secondary to their inherent endoperoxidase activity.^3^, ^4^, ^5^, ^6^, ^7^, ^8^, ^9^, ^10^ ARS compounds could exert tumouricidal activity in multiple types of tumours, such as hepatocellular carcinoma, breast cancer, prostate cancer and ovarian cancer,^4^, ^7^, ^8^, ^11^, ^12^, ^13^, ^14^, ^15^ and potentially regulate cell growth, apoptosis, the cell cycle and invasion.^5^, ^7^, ^8^, ^12^ Interestingly, ARS compounds could induce cell autophagy in ovarian cancer^12^ and enhance the antitumour immune response of T cells.^4^ Based on the regulation of autophagy by ARS compounds and the close link between autophagy and metabolism, we propose that ARS compounds regulate the progression of cancer at least partially by reprogramming cancer cell metabolism.

HMGCR (3‐hydroxy‐3‐methylglutaryl coenzyme A reductase), the rate‐limiting enzyme and key regulator of the mevalonate (MVA) pathway, which is responsible for the production of cholesterol, isoprenoids and ubiquinone,^16^ is tightly regulated by SREBP2 (sterol regulatory element‐binding protein 2).^17^ Multiple studies have shown that HMGCR and the MVA pathway can promote tumourigenesis.^18^, ^19^, ^20^, ^21^ Furthermore, as an HMGCR inhibitor,^22^ statin is also deemed an inhibitor of carcinogenesis.^23^, ^24^, ^25^ Dysregulation of the MVA pathway is commonly observed in glioma, and the related FDPS (farnesyl diphosphate synthase) gene was identified as a new metabolic oncogene and a therapeutic candidate for glioblastoma treatment.^26^ Additionally, *myc* has been shown to mediate its oncogenic effects on glioma tumour‐initiating cells partially by affecting MVA metabolism.^27^ Therefore, targeting the MVA pathway would be beneficial to the treatment of glioma.

Senescence is one of the most common mechanisms that cells employ to eliminate damage and inhibit cell proliferation.^28^ Senescence is particularly relevant in ageing and cancer, both of which are characterized by severe cellular damage accumulation. Senescence can be induced by various cellular stimuli, many of which involve the activation of p53 and its consequential activation of cyclin‐dependent kinase (CDK) inhibitors, such as p16 (also known as INK4A), p15 (also known as INK4B), p21 (also known as WAF1) and p27.^29^, ^30^ Therefore, senescence is becoming a promising treatment to combat the progression of cancer.^31^, ^32^

In this work, we investigated the anticancer effects of artesunate (ART), the most soluble and effective ARS derivative, on glioma and demonstrated its underlying regulation of cancer metabolism and senescence.

## 2 MATERIALS AND METHODS

### 2.1 Compounds and antibodies

Artesunate (ART), purchased from Xi'an HaoYuan Bio Technology Co., Ltd., had a purity of 99.86% and was dissolved in ddH_2_O for this study.

Antibodies against GAPDH (#5174), P53 (#2524), Flag‐tag (#14793) and myc tag (#2276) were purchased from Cell Signaling Technology, Inc. Antibodies against HMGCR (HPA008338) and LDHA (SAB2108638) were obtained from Sigma‐Aldrich, Inc. Antibodies for ENO1 (ab155102), HK2 (ab104836) and SREBP2 (ab30682) and all secondary antibodies (anti‐mouse, anti‐goat and anti‐rabbit immunoglobulin G) were purchased from Abcam.

### 2.2 Cell culture

Human glioma cell lines (U251, U87, U138 and SK‐N‐SH) originally obtained from the American Type Culture Collection (ATCC) were purchased from the Shanghai Cell Bank of the Chinese Academy of Science (Shanghai, China) and cultured in DMEM (Invitrogen‐Gibco Co.) supplemented with 10% FBS (Gibco) and antibiotics at 37°C and 5% CO_2_ according to the ATCC instructions. Cell transfection was performed using Lipofectamine 2000 (Thermo Fisher, #11668027) according to the manufacturer's instructions.

### 2.3 Crystal violet assay

Cells were seeded at 1000 cells/well in a 24‐well plate. Seven days later, the medium was removed, and the cells were fixed with methanol and then stained with a crystal violet solution (0.5%) for 5 minutes. Finally, the cells were washed with PBS and photographed.

### 2.4 Apoptosis assay

An annexin V‐FITC/PI kit (Invitrogen) was employed to detect cell apoptosis. Briefly, cells were collected, washed and resuspended in binding buffer (100 μL); then, 5 μL of Annexin V‐FITC and 1 μL of PI were added for staining. After 15 minutes of incubation at room temperature, the cells were analysed using a FACScan instrument (Beckman Coulter).

### 2.5 Wound healing assay

Cells were scraped in a straight line with a 100‐µL pipette tip after growing to 90% confluence in dishes. Twenty‐four hours later, wound healing was examined and photographed under a light microscope.

### 2.6 Soft agar assay

Low‐melt agarose (0.6%) purchased from Solarbio was coated onto 24‐well plates and allowed to solidify at 4°C. Cells were mixed with 0.35% low‐melt agarose and seeded at 500 cells/well density in the wells. The colonies were photographed after 2 weeks of incubation at 37°C, and the number of colonies was counted under a light microscope.

### 2.7 Distant seeding assay

U251 and U87 cells were labelled with the luciferase gene and selected with puromycin. The cells with resistance were pooled and injected into nude mice (BALB/c, nu/nu, 6 weeks) through their tail veins (10^6^ per mice). Artemisinin was administered three times per week at a dose of 50 mg/kg starting the next day. The distant seeding of the U251 and U87 cells was monitored using an in vivo imaging system (Xenogene), and the photon counts were calculated. All animal experiments were approved by the Shenzhen University biomedical ethics committee.

### 2.8 Immunohistochemistry

Tissues were first fixed in 10% formaldehyde overnight at room temperature, embedded in paraffin and then cut into 4‐μm‐thick sections. Next, the sections were incubated at 60°C for 1 hr, deparaffinized in xylene and rehydrated with gradient ethanol solutions. Antigen retrieval was achieved using sodium citrate buffer (10 mmol/L, pH 6.0; with 0.05% Tween 20) and boiling for 10 minutes in a microwave. All endogenous peroxidase activity was blocked by 20 minutes of treatment with 3% hydrogen peroxide in methanol. The slides were then blocked with 10% normal serum supplemented with 1% BSA in TBS for 2 hours at room temperature and incubated with the indicated primary antibody at 4°C overnight. Then, the sections were sequentially incubated with a secondary antibody, biotinylated HRP‐coupled anti‐rabbit IgG (1:500, Proteintech) and 3′,3‐diaminobenzidine for 1 hours each at 37°C. Cell nuclei were visualized by counterstaining with haematoxylin.

### 2.9 qRT‐PCR

Total RNA was extracted via TRIzol reagent (Invitrogen), and reverse transcription was performed immediately using a PrimeScript™ RT Reagent Kit with gDNA Eraser (Perfect Real Time; TaKaRa Bio). Then, SYBR^®^ Premix Ex Taq (RR420A, TaKaRa Bio) was employed for q‐PCR analysis in a 20‐μL reaction system according to the manufacturer's instructions. Each sample was amplified in triplicate and processed on an Applied Biosystems real‐time PCR machine (7500, Foster City, CA, USA). The fold change in gene expression was analysed using an I7500 real‐time detection system (Applied Biosystems). The primers used for qRT‐PCR were as follows: HMGCR: F: TGATTGACCTTTCCAGAGCAAG, R: CTAAAATTGCCATTCCACGAGC; P21: F: TGTCCGTCAGAACCCATGC, R: AAAGTCGAAGTTCCATCGCTC; and beta‐actin: F: CATGTACGTTGCTATCCAGGC, R: CTCCTTAATGTCACGCACGAT.

### 2.10 Immunofluorescence

Cells were seeded on cover glass; upon reaching 90% confluence, the cells were washed with PBS and fixed for 10 minutes in ice‐cold methanol. After washing three times with PBS, the cells were blocked with 5% BSA for 1 hour at 37°C and incubated with a primary antibody against SREBP2 at 4°C overnight. The next day, the cells were incubated with a FITC‐coupled secondary antibody for 1 hour at 37°C. After sufficient PBS washing, the cells were then stained with Hoechst solution and examined under a confocal microscope.

### 2.11 GST pull‐down assay

The P53 coding sequence was amplified by reverse transcription PCR and then subcloned into the pGEX4T1 vector (Amersham Pharmacia Biotech). The GST fusion protein was purified using Sepharose 4B beads (GE Healthcare) according to the kit instructions. Then, GST fusion protein (10 µg) was incubated with the cell lysates overnight, after which Sepharose 4B beads were added for another 4‐hour incubation period. Finally, the beads were washed, the immunoprecipitants were eluted with loading buffer, and the proteins were examined by Western blot.

### 2.12 Immunoprecipitation

Cells were transfected with the Flag‐SREBP2N and myc‐P53 plasmids using Lipofectamine 2000. Forty‐eight hours later, the cells were harvested with RIPA buffer. After centrifugation, the supernatants were incubated with Flag antibody‐coupled beads for 4 hours at 4°C. The beads were then washed with RIPA buffer, the immunoprecipitants were eluted with loading buffer, and the target proteins were examined by Western blot.

### 2.13 Senescence assay

Senescence was evaluated through SA‐β‐Gal activity analysis with a Senescence Detection Kit (BioVision). In brief, cells were fixed at 70% confluence according to the manufacturer's instructions and then incubated at 37°C overnight with a staining solution containing X‐gal substrate. The cells were then observed under a microscope (Olympus BX40) for blue staining.

## 3 RESULTS

### 3.1 Artesunate inhibited the growth, migration and anchorage‐independent growth of glioma cells

**Figure 1:**
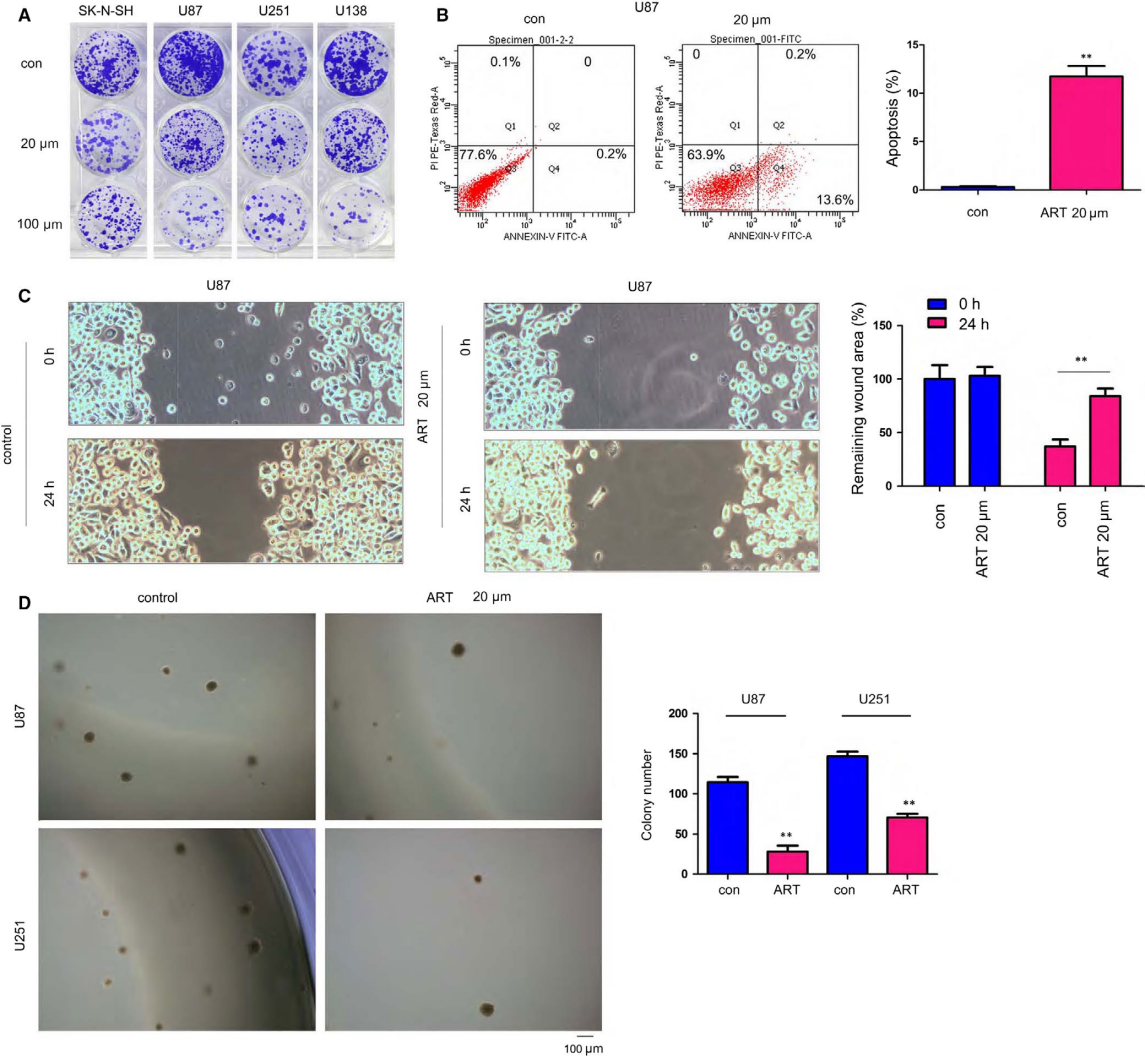
Artesunate inhibited glioma cell growth. A, The effects of artesunate on glioma cell growth were assessed by crystal violet staining. U87, U251, U138 and SK‐N‐SH cells were seeded in a 24‐well plate at a density of 1000 cells/well. Cells were treated with 20 µm or 100 µm artesunate for 14 d. Then, the cells were stained with a crystal violet solution. B, The effects of artesunate on glioma cell apoptosis were assessed by Annexin V staining, and the results were quantified. C, The effects of artesunate on glioma cell motility were assessed with a wound healing assay, and the results were quantified. D, The effects of artesunate on the anchorage‐independent growth of glioma cells were assessed with a soft agar assay. Scale bar, 100 µm. *P < .05; **P < .01

To study the therapeutic effects of ART on glioma, we first examined whether ART could influence glioma cell growth, migration and anchorage‐independent growth. As shown by the crystal violet assay, ART inhibited the growth of SK‐N‐SH, U87, U251 and U138 cells (Figure 1A). For further confirmation, we assessed the effects of ART on glioma cell apoptosis (Figure 1B). Treatment with ART dramatically induced U87 cell apoptosis. Moreover, ART effectively impaired the motility of U87 cells in a wound healing assay (Figure 1C). In addition, in a soft agar assay, ART dramatically inhibited the anchorage‐independent growth of U87 and U251 cells (Figure 1D). Taken together, our observations suggested that ART could effectively inhibit glioma cell malignancy.

### 3.2 Artesunate inhibited HMGCR expression in the mevalonate (MVA) pathway

**Figure 2:**
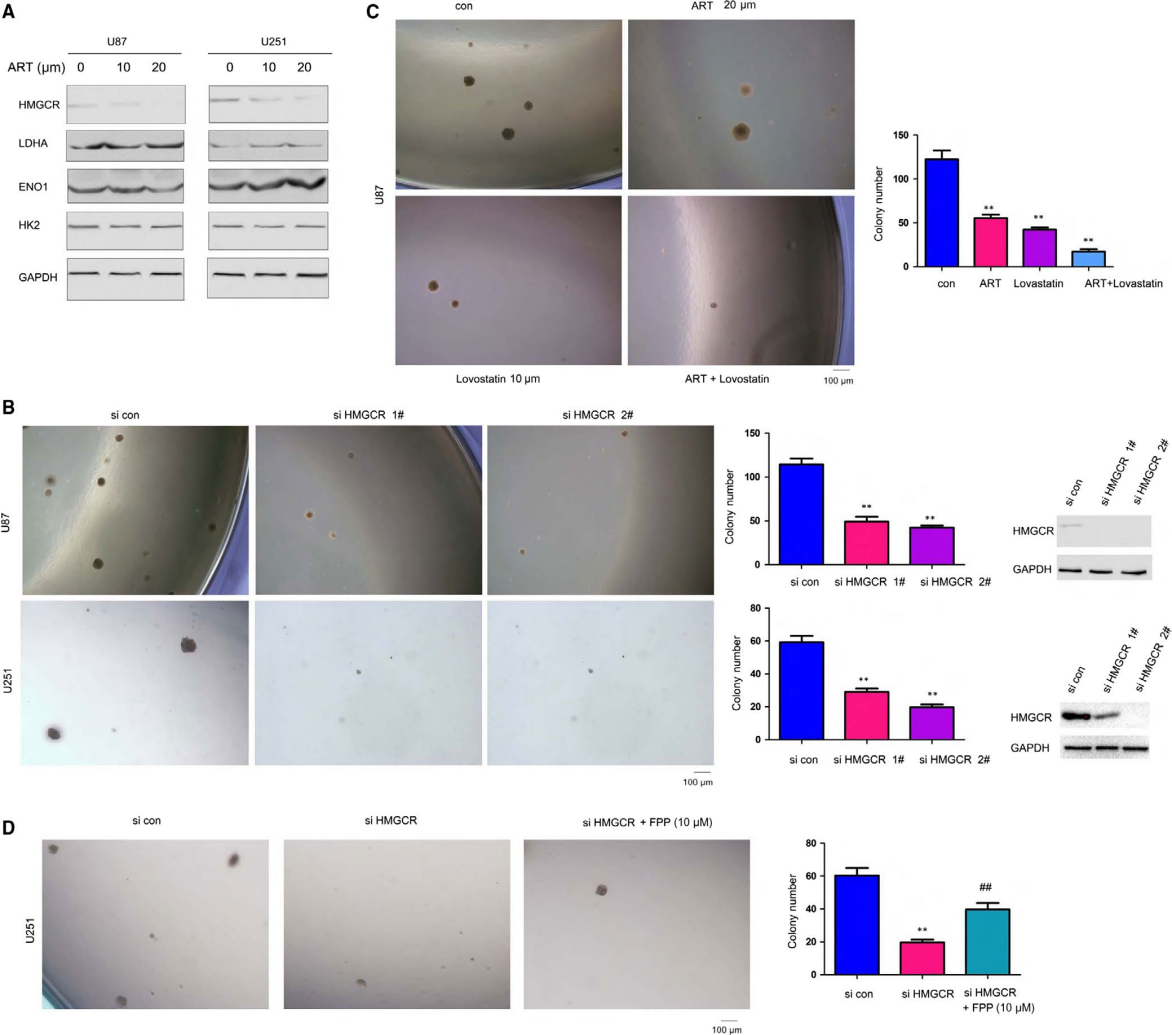
Artesunate inhibited HMGCR expression. A, The expression of HMGCR in U87 and U251 cells upon treatment with artesunate was examined. B, A soft agar assay was performed using HMGCR knockdown U87 and U251 cells. C, A soft agar assay was performed using artesunate‐ and/or lovastatin‐treated U87 cells. D, A soft agar assay was performed to examine the rescue effects of FPP. Scale bar, 100 µm. *P < .05; **P < .01

Although several studies have shown that ART inhibits the malignant actions of glioma cells, the molecular mechanism is poorly understood. To elucidate the mechanism, we investigated whether ART could inhibit the expression of enzymes involved in glycolysis and cholesterol synthesis (eg, the MVA pathway). Treatment with ART reduced the expression of HMGCR, the rate‐limiting enzyme of the MVA pathway. However, ART exerted little effect on the expression of enzymes involved in glycolysis (ENO1, LDHA and HK2) (Figure 2A). We next evaluated the roles of HMGCR in the progression of glioma and found that HMGCR knockdown could inhibit the anchorage‐independent growth of U87 and U251 cells (Figure 2B), which actually mimicked the phenotype induced by ART treatment (Figure 1D). Furthermore, lovastatin, an inhibitor of HMGCR, acted synergistically with ART to inhibit the anchorage‐independent growth of U87 cells (Figure 2C). However, the inhibitory effects on the anchorage‐independent growth by down‐regulation of HMGCR were rescued by FPP, the downstream products of HMGCR (Figure 2D). Taken together, these data suggested that ART inhibited the anchorage‐independent growth of glioma cells by negatively regulating the MVA pathway.

### 3.3 Artesunate inhibited the distant seeding of U251 and U87 cells in vivo

**Figure 3:**
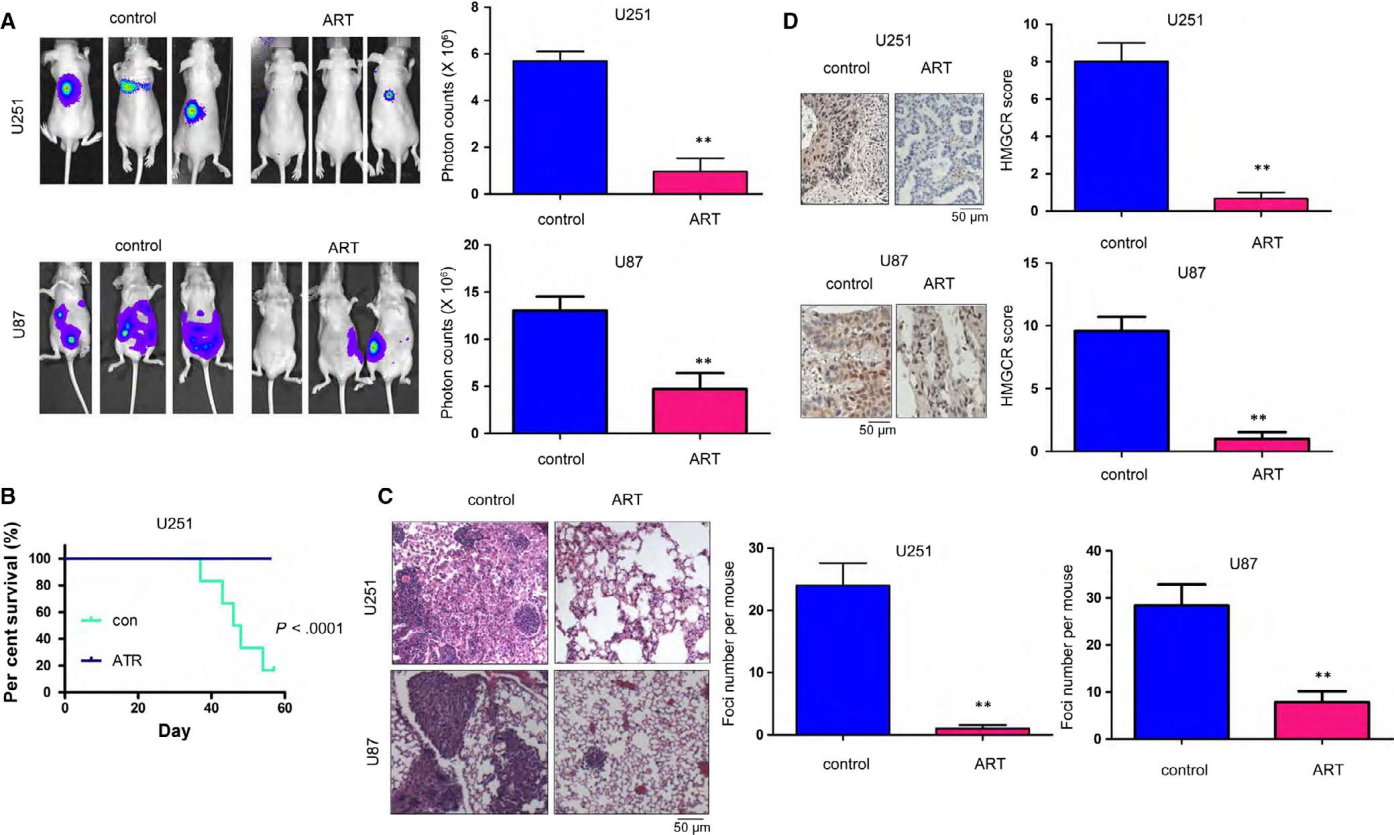
The distant seeding of U251 and U87 cells was inhibited by artesunate. A, The effects of artesunate on distant seeding were evaluated by an in vivo image system. U251 and U87 cells were labelled with luciferase and injected into mouse tail veins. The mice were treated with artesunate (50 mg/kg) every other day. The distant seeding of the U251 and U87 cells was examined using an in vivo image system. B, The effects of artesunate on the survival of nude mice injected with U251 and U87 cells were examined. C, HE staining of the lungs of mice injected with U251 cells and treated with artesunate. D, IHC analysis was performed to examine the expression of HMGCR in the lungs of mice injected with U251 and U87 cells and treated with artesunate. Scale bar, 50 µm. *P < .05; **P < .01

Metastasis of glioma cells to the lungs is very common. Therefore, we next evaluated the therapeutic effects of ART on distant seeding in a mouse model. Glioma U251 and U87 cells were labelled using a luciferase gene, which enabled the tracing of U251 and U87 cells using an in vivo image system and luciferin administration, a substrate of luciferase. As shown in Figure 3A, administration of ART impaired the distant seeding (demonstrated as the signal intensity) of U251 and U87 cells that were injected into nude mice via their tail vein. Consistent with these observations, ART also improved the survival of nude mice injected with U251 cells via their tail vein (Figure 3B). Additionally, administration of ART decreased the number of metastatic foci formed in the lung (Figure 3C). Consistent with the data shown in Figure 2A, HMGCR expression was nearly diminished in the lungs of mice treated with ART (Figure 3D). In summary, these results clearly demonstrated the antimetastatic activity of ART.

### 3.4 Artesunate inhibited the nuclear localization and transcriptional activity of SREBP2

**Figure 4:**
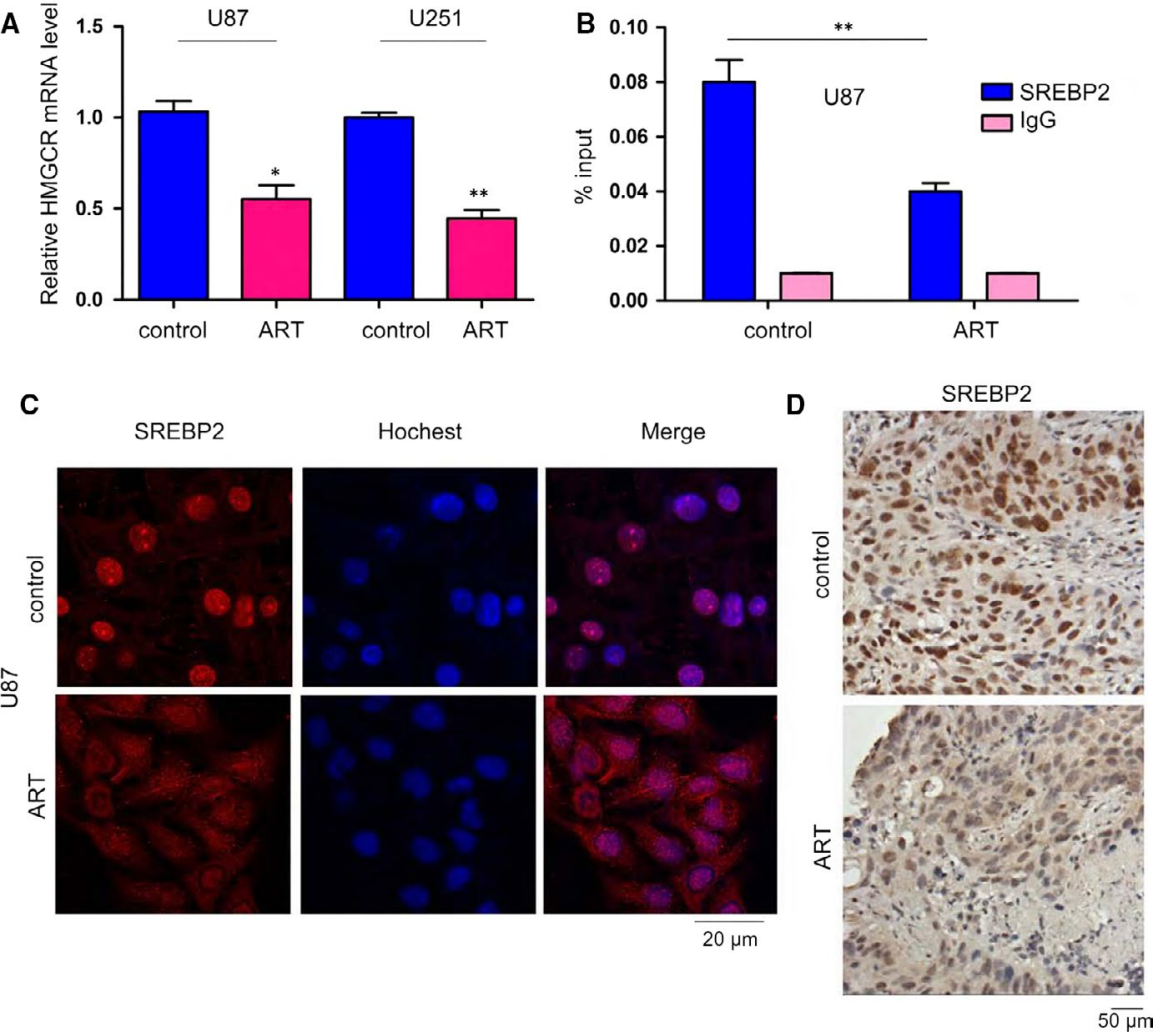
Artesunate inhibited the nuclear localization of SREBP2. A, q‐PCR was performed to examine the effects of artesunate on the mRNA expression of HMGCR. B, A ChIP assay was performed to examine the effects of artesunate on the binding of SREBP2 to the HMGCR promoter. C, Immunofluorescence staining was performed to examine the effects of artesunate on the nuclear localization of SREBP2 in U87 cells; scale bar, 20 µm. D, IHC staining was performed to examine the effects of artesunate on the nuclear localization of SREBP2 in distant seeded metastatic foci; scale bar, 50 µm. *P < .05; **P < .01

The decreased expression of HMGCR in ART‐treated U251 and U87 cells prompted us to investigate the intrinsic mechanisms. We first examined the effects of ART on the mRNA levels of HMGCR in U87 and U251 cells and found that ART‐treated cells had lower HMGCR mRNA levels than their counterpart cells (Figure 4A), suggesting the possible transcriptional regulation of HMGCR by ART. Moreover, ChIP assay results demonstrated that ART effectively inhibited the binding of SREBP2 to the HMGCR promoter (Figure 4B). Consistent with these observations, SREBP2 showed little nuclear localization in U87 cells treated with ART (Figure 4C). Additionally, the U87 xenografts treated with ART showed less nuclear‐localized SREBP2 than those un‐treated counterparts (Figure 4D). Collectively, these results suggested that ART impaired the transcriptional activity of SREBP2.

### 3.5 Artesunate disrupted the SREBP2‐P53 interaction, induced P21 expression and promoted senescence in glioma cells with wild‐type P53

**Figure 5:**
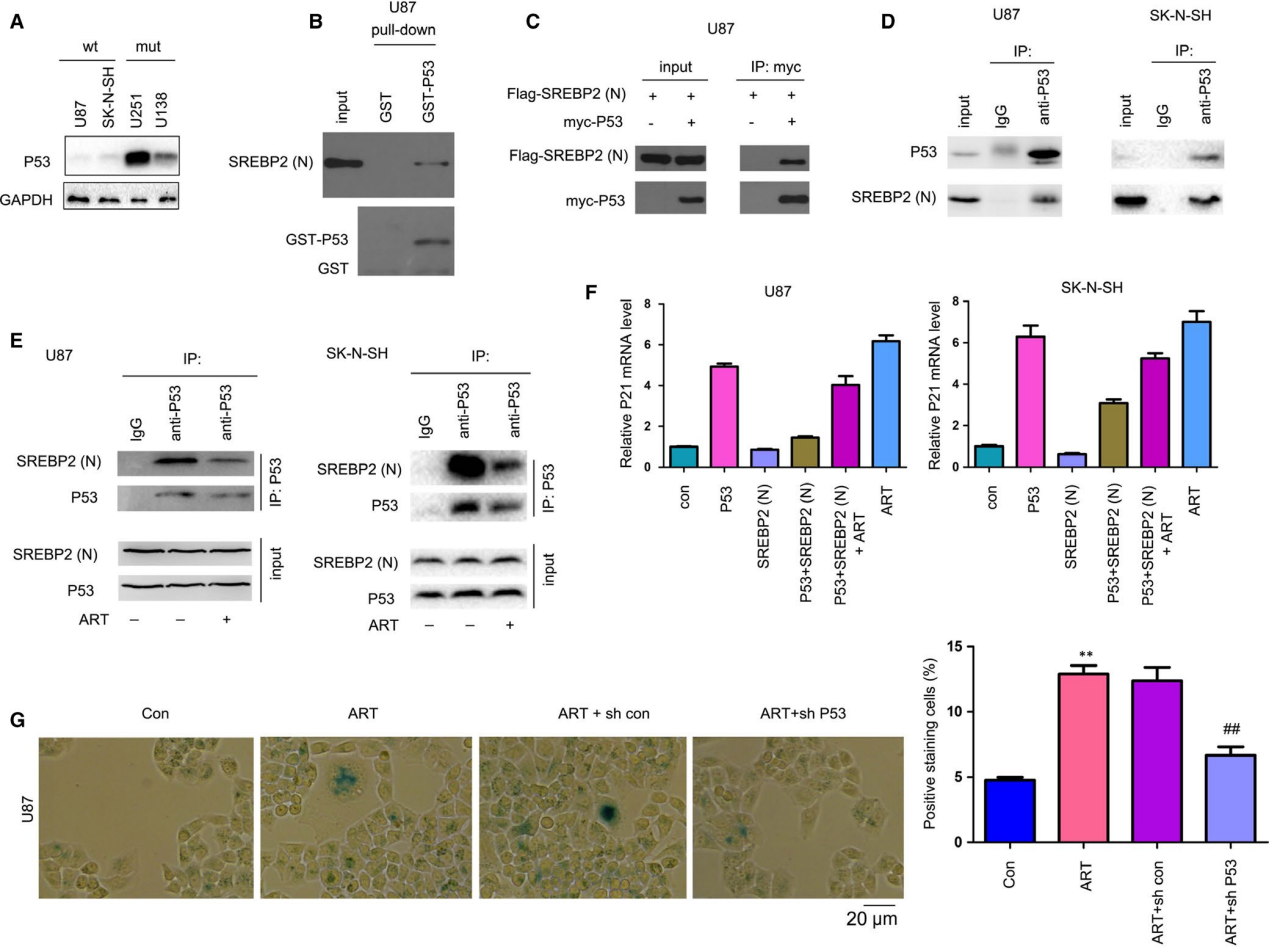
Artesunate disrupted the interaction between SREBP2 and P53. A, The P53 protein levels were examined by Western blot. B, The interaction between the N‐terminus of SREBP2 and P53 was examined by a GST pull‐down assay. The fusion protein GST‐P53 was purified and incubated with U87 cell lysates. C, An immunoprecipitation assay was performed to examine the interaction between exogenous Flag‐tagged SREBP2(N) and myc‐tagged P53. D, An immunoprecipitation assay was performed to examine the interaction between endogenously expressed SREBP2(N) and P53. E, The effects of artesunate on the interaction between SREBP2N and P53. F, The effects of artesunate and SREBP2N on the mRNA levels of P21 were examined by q‐PCR. G, The effects of artesunate on the senescence of glioma cells were examined by measuring SA‐β‐Gal activity; scale bar, 20 µm. *P < .05; **P < .01

It was reported that SREBP1 can interact with mutant P53 and activate the MVA pathway. However, the roles of SREBPs in P53‐mediated transcription remain unknown. Therefore, we evaluated the roles of SREBP2 in P53‐mediated functions. Firstly, the protein levels of P53 in four glioma cell lines were examined. As shown in Figure 5A, U87 and SK‐N‐SH, which harboured wild‐type P53, showed very lower P53 expression, while U251 and U138, which harboured mutant P53, showed higher P53 expression. In a GST pull‐down assay, the fusion protein GST‐P53 could pull down SREBP2 (Figure 5B). Additionally, the exogenously expressed Flag‐SREBP2 and myc‐P53 formed a complex in an immunoprecipitation assay (Figure 5C). Consistently, endogenous SREBP2 and P53 interacted with each other in U87 and SK‐N‐SH cells (Figure 5D). However, ART disrupted the interaction between SREBP2 and P53 in the immunoprecipitation assay (Figure 5E). P21 has been reported to be the target gene of P53. As shown in Figure 5F, SREBP2 inhibited the P21 mRNA expression induced by P53, which was restored by ART (Figure 5F). P21 is a regulator of cell senescence. Consistently, ART induced senescence, which was abolished by P53 knockdown (Figure 5G). In summary, ART disrupted the SREBP2‐P53 interaction, induced P21 expression and promoted senescence in glioma cells.

## 4 DISCUSSION

**Figure 6:**
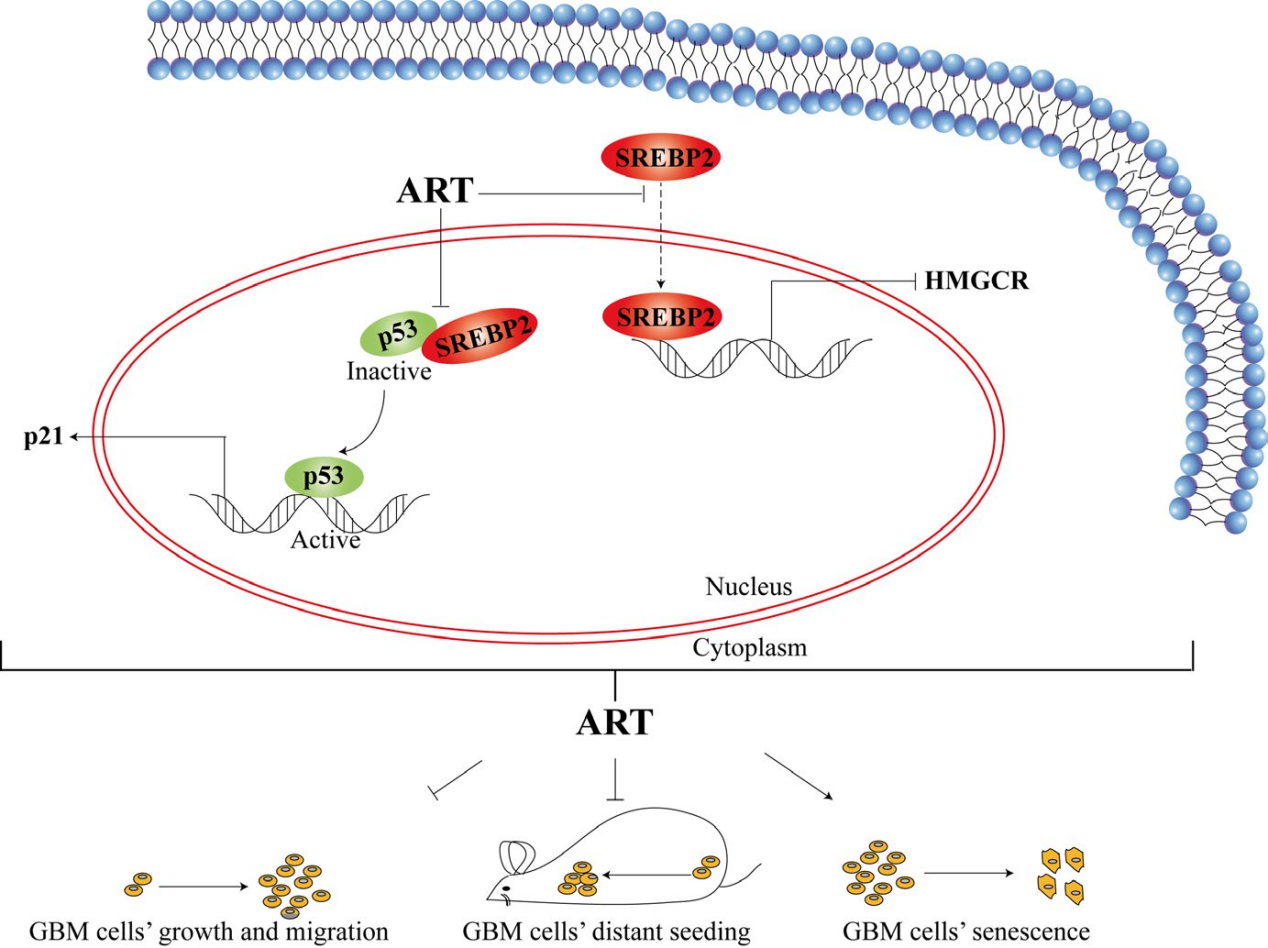
Schematic of the proposed mechanism(s) of artesunate (ART) action. ART inhibits glioma by inhibiting SREBP2 nuclear localization and, in turn, the expression of its target gene HMGCR and/or by disrupting the SREBP2 and P53 interaction to up‐regulate P21 and induce senescence

Our group has long focused on studying the anticancer effects of ARS compounds on different types of cancer, including hepatocellular carcinoma, breast cancer and ovarian cancer.^6^, ^7^, ^8^, ^9^, ^10^ Several other studies have also shown that ARSs promote cell apoptosis and inhibit invasion in glioma,^33^ and the mechanisms involve AKT signalling^34^ and ROS‐β‐catenin signalling.^35^ However, the roles of ARS compounds in cancer metabolism have never been elucidated. In the present study, we demonstrated that the ARS analogue ART could inhibit glioma malignancy through two mechanisms: (a) inhibiting the nuclear localization of SREBP2 and its target gene HMGCR, which have been demonstrated to be oncogenes, and (b) disrupting the interaction between SREBP2 and P53, which up‐regulated P21 expression and induced senescence (Figure 6). Our studies suggested novel anticancer behaviours(s) of ARSs for glioma therapy.

Artemisinin and its derivatives, especially ART, are reported to play a role in the prevention or treatment of diabetes, mainly by inducing protective IL‐4‐producing T cells and regulatory T cells, suggesting that ART might inhibit cancer progression by reprogramming the metabolic profile of cancer cells.^36^ The present study showed that ART could decrease HMGCR protein levels and inhibit the MVA pathway. As HMGCR has been reported to be an oncogene in breast and pancreatic cancer and up‐regulated in multiple cancer types,^20^, ^37^ we speculated that ART could inhibit the progression of multiple cancer types, at least partially, by down‐regulating HMGCR expression.

One of the most interesting findings in our study is the physiological and functional interaction between P53 and SREBP2. SREBP1 has been reported to interact with mutant P53, and mutant P53 cooperates with SREBP1 to activate the MVA pathway in breast cancer.^38^ However, whether and how SREBP2 could affect the functions of P53 remains unknown. The present study revealed the negative regulation of P53 and the inhibition of senescence by SREBP2, suggesting the mutual regulation of these proteins. Moreover, ART could abolish the interaction and regulation of SREBP2 and P53 and induce senescence, which provides a good explanation for the anticancer activity of ARS compounds.

Taken together, our results have demonstrated a novel therapeutic effect of ART on glioma cells. For the first time, we elucidated that ARS compounds can influence cancer metabolism, which absolutely provides promising inspiration for further clinical exploration for cancer treatment.

## CONFLICT OF INTEREST

The authors confirm that there are no conflicts of interest.

## AUTHOR CONTRIBUTIONS

YN and TC designed the study. SW, LL and YL performed the experiments with the assistance of WY and QO. SW, ZC and LL analysed the data. SW, ZC and FZ drafted the manuscript. YN and TC revised the manuscript and supervised the work.

## Data Availability

The data that support the findings of the study are available from the corresponding author upon reasonable request.
